# Neutrophils Play an Important Role in the Recurrence of Chronic Rhinosinusitis with Nasal Polyps

**DOI:** 10.3390/biomedicines10112911

**Published:** 2022-11-12

**Authors:** Hyunjae Yu, Dong-Kyu Kim

**Affiliations:** 1 Institute of New Frontier Research, Division of Big Data and Artificial Intelligence, Hallym University College of Medicine, Chuncheon 24253, Korera; 2 Department of Otorhinolaryngology-Head and Neck Surgery, Chuncheon Sacred Heart Hospital, Hallym University College of Medicine, Chuncheon 24253, Korea

**Keywords:** neutrophil, rhinosinusitis, sinusitis, nasal polyp, machine learning

## Abstract

Despite the heterogeneity of chronic rhinosinusitis (CRS), a clear link exists between type 2 immunity and the severity of CRS with nasal polyps (CRSwNP). However, recent studies have demonstrated that patients with severe type 2 CRSwNP also display abundant neutrophilic inflammation. Therefore, we investigated the factors associated with the recurrence of CRSwNP following sinus surgery using a machine-learning algorithm. We collected the demographics, clinical variables, and inflammatory profiles of 210 patients with CRSwNP who underwent sinus surgery. After one year, we evaluated whether each patient showed recurrence. Machine-learning methods, such as decision trees, random forests, and support vector machine models, have been used to predict the recurrence of CRSwNP. The results indicated that neutrophil inflammation, such as tissue and serum neutrophils, is an important factor affecting the recurrence of surgical CRSwNP. Specifically, the random forest model showed the highest accuracy in detecting recurrence among the three machine-learning methods, which revealed tissue neutrophilia to be the most important variable in determining surgical outcomes. Therefore, our machine-learning approach suggests that neutrophilic inflammation is increased in patients with difficult-to-treat CRSwNP, and the increased presence of neutrophils in subepithelial regions is closely related to poor surgical outcomes in patients with CRSwNP.

## 1. Introduction

Chronic rhinosinusitis (CRS) is one of the most common chronic inflammatory diseases and is characterized by local inflammation of the upper airways. Currently, CRS is divided into two main phenotypes based on nasal endoscopy: CRS with nasal polyps (NP) (CRSwNP) and CRS without NP (CRSsNP); however, CRS is a broad syndrome characterized by many features in individuals [1]. Generally, CRSwNP is more severe than CRSsNP and is usually treated with sinus surgery and medical treatment. However, despite repeated sinus surgeries combined with aggressive medical therapy, in some cases tend to be poorly controlled with high recurrence rates. Several previous studies have reported that despite surgery plus appropriate medical therapy, nearly 50% of the patients showed NP recurrence after one year [2,3,4].

Recently, increasing evidence has suggested that inflammation in CRS is highly heterogeneous and is divided into three main endotypes: (1) type 1 immune response with type 1 cytokine (interferon-γ) elevation, (2) type 2 immune response with eosinophilia and type 2 cytokine (interleukin [IL]-4,-5,-13) elevation, and (3) type 3 immune response with neutrophilia and type 3 cytokine (IL17A) elevation [5,6,7]. Neutrophilic infiltration is attributed to the reaction of epithelial cells stimulated by Charcot-Leyden crystals (CLC), which are degradation byproducts of eosinophils [8]. Thus, severe neutrophilic inflammation could be associated with a severe eosinophilic type 2 immune response in CRSwNP, independent of IL17A [9]. In addition, a previous study described how the B-cell activating factor is associated with refractoriness in CRSwNP via a Th17 immune response and neutrophilic recruitment [10]. Other studies also demonstrated that tissue neutrophilia is an important factor associated with treatment outcomes in patients with CRSwNP [11,12,13].

However, which factors, including neutrophils, are the most important in the refractoriness of CRSwNP remains unclear. Currently, machine-learning models are widely used in several different fields, from finance to healthcare, for detecting major variables. Therefore, in this study, we attempted to identify the effects of important predictor variables on the recurrence of CRSwNP following sinus surgery with medical treatment using modern machine-learning algorithms and methods.

Recently, the machine-learning approach has enabled computational models composed of multiple processing layers to predict data with multiple levels of abstraction [14,15]. Thus, numerous researchers have begun to focus on machine learning as a promising technology to solve major problems in various clinical fields [15,16,17]. Therefore, in this study, we investigated the factors affecting the surgical outcomes of patients with CRSwNP using a machine-learning algorithm with a focus on neutrophilic localization. We speculated that applying a machine-learning algorithm could provide new insights, helping us further understand the role of neutrophils in the pathogenesis of CRSwNP.

## 2. Materials and Methods

### 2.1. Patients and Tissue Samples

This study was conducted in accordance with the guidelines of the Declaration of Helsinki and approved by the Institutional Review Board (IRB) of the Hallym Medical University Chuncheon Sacred Hospital (Chuncheon, Korea, IRB No. 2019-10-009). All the participants provided written informed consent prior to the study. CRS diagnosis was made based on the history, physical examination, nasal endoscopic examination, and computed tomography (CT) findings of the sinuses according to the 2020 European Position Paper on Rhinosinusitis and Nasal Polyps (EPOS) guidelines [1]. The exclusion criteria were as follows: age younger than 18 years; history of receiving treatment with antibiotics, systemic or topical corticosteroids, or other immune-modulating drugs during the two weeks prior to surgery; and diagnosis with unilateral rhinosinusitis, antrochoanal polyp, allergic fungal sinusitis, cystic fibrosis, or immotile ciliary disease. Tissue samples from NP were obtained from patients with CRSwNP during routine endoscopic sinus surgery. The CRSwNP endotype was defined according to the histopathological findings of hematoxylin and eosin-stained tissue samples (eosinophilic NP: >10% eosinophils per high-power field; non-eosinophilic NP: ≤10% eosinophils per high-power field) [14]. All the enrolled patients were also classified into subgroups according to the algorithm of the Japanese Epidemiological Survey of Refractory Eosinophilic Chronic Rhinosinusitis study [15]. Subgrouping was performed by considering several clinical factors, including bilateral disease sites, NP, sinus CT findings, eosinophilia in the peripheral blood, and comorbidities (bronchial asthma and aspirin-exacerbated respiratory disease/nonsteroidal anti-inflammatory drug-exacerbated respiratory disease). The atopic status of the study participants was evaluated using the ImmunoCAP^®^ assay (Thermo Scientific Inc., Waltham, MA, USA) to detect immunoglobulin E antibodies against six common aeroallergens (house dust mites, molds, trees, weeds, grass pollen, and animal dander). A patient with asthma was defined as one who exhibited chronic airway symptoms (dyspnea, cough, wheezing, or sputum), reversible airflow limitations, an increase in the forced expiratory volume of ≥12% or 200 mL in 1 s after using a bronchodilator, or a methacholine provocation test result of PC20 ≤16 mg/mL. Disease severity was evaluated using CT images based on the Lund-Mackay (LM) CT scoring system. LM CT scores, global osteitis scores, and olfactory CT scores were calculated on the preoperative CT scans.

### 2.2. Cytokine Measurement

The protein concentrations in the tissue extracts were determined using the Pierce 660 nm Protein Assay Kit (Thermo Scientific Inc.), and the samples were thawed at room temperature and vortexed for thorough mixing. Tissue homogenates were assayed for periostin proteins using commercially available enzyme-linked immunoassay kits (R&D Systems, Minneapolis, MN, USA). Multiple cytokine analysis kits were obtained from R&D Systems, and data were collected using a Luminex 100 (Luminex, Austin, TX, USA). Data analysis was performed using MasterPlex QT (version 2.0; MiraiBio, Alameda, CA, USA). All the assay procedures were performed in duplicate, according to the manufacturer’s protocol. All the protein levels in the tissue homogenates were normalized to the total protein concentration.

### 2.3. Statistical Analysis

To identify and rank the important factors influencing the recurrence of CRSwNP, we randomly divided the enrolled patients into a training set (two-thirds of the patients) and a validation set (one-third of the patients) using a random number generator without stratification. The prediction models were designed and implemented using machine learning. In this study, we constructed three machine-learning prediction models: (1) decision tree (DT), (2) random forest, and (3) support vector machine (SVM). A DT is a type of supervised machine learning in which data are continuously split according to a certain parameter. On the DT, we created a training model that could predict the class or value of the target variable by learning simple decision rules inferred from the training dataset. Additionally, we examined the performance of the training model using the test dataset. Thus, each node in the DT represents a feature of an instance to be classified, and each branch represents a value that the node can assume. Random forest is an ensemble-type classification method created using bootstrap samples of the training data and random feature selection in tree induction. Thus, it is one of the supervised learning consisting of multiple DTs. The SVM is also a supervised machine-learning algorithm based on a statistical learning theory using the concept of structural risk minimization. It solves binary classification problems by fitting a maximum margin discriminator to a dataset in kernel-induced feature space. Thus, it could detect the optimal hyperplane, which can classify new data. Additionally, we calculated the receiver operating characteristic (ROC) curve area under the ROC curves (AUC), positive/negative predictive value, accuracy, sensitivity, specificity, and F1 score. Statistical analyses were performed using R version 3.4.2 (R Foundation for Statistical Computing, Vienna, Austria) and GraphPad Prism (version 7.0; GraphPad Software Inc., La Jolla, CA, USA) software.

## 3. Results

**Figure 1:**
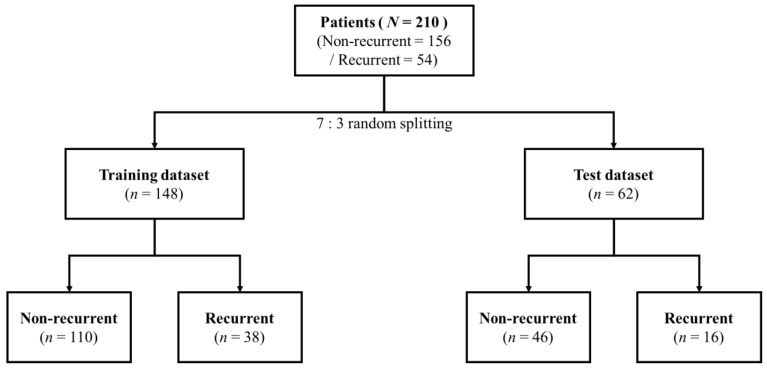
Study design. Patients were randomly divided into training and test sets (2/3 vs. 1/3 of patients).

Patient characteristics are presented in Table 1. Endotype, sex, age, asthma history, LM score, GOS score, sinus dominance, olfactory CT score, tissue eosinophilia, HNE subepithelial count, HNE perivascular count, serum eosinophil, IL5, IL6, INFγ, TNFɑ, and IL10 saw significant differences between the two groups. We evaluated the disease control status of individual patients one year after endoscopic sinus surgery. The patients were classified into non-recurrence and recurrence (partly controlled plus uncontrolled) groups according to the EPOS guidelines [1], considering the presence and severity of the four major sinonasal symptoms, sleep disturbance (or fatigue), nasal endoscopic evaluation, and the need for oral medication. The patients were then randomly divided into training and test sets (Figure 1). The profiles of each dataset are presented in Table 2 and Table 3.

### Prediction of Surgical Outcomes for CRSwNP

**Figure 2:**
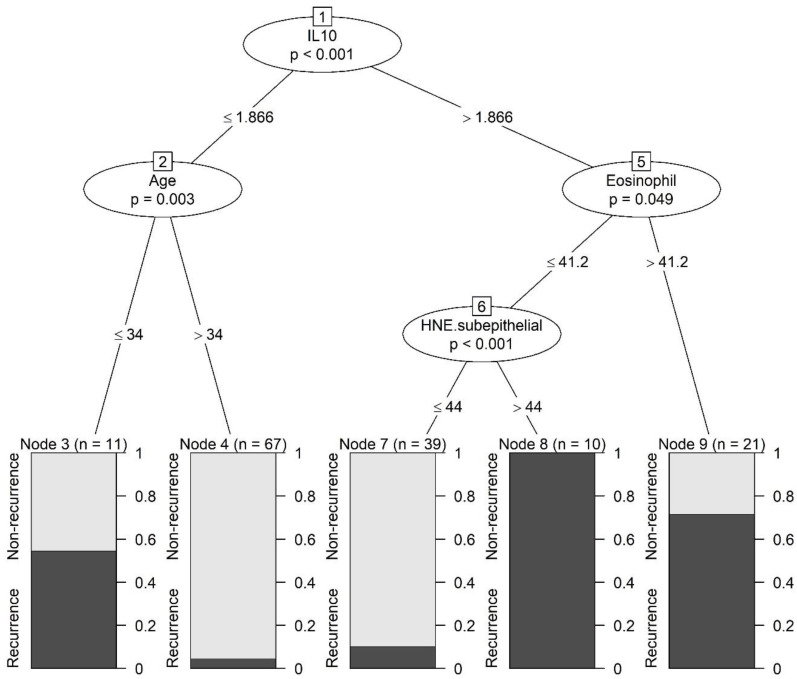
Result of the decision tree model for the evaluation of surgical outcomes in patients with chronic rhinosinusitis with nasal polyps. HNE—human neutrophil elastase; IL—interleukin.

To investigate the factors influencing the surgical outcomes of patients with CRSwNP, we employed three machine-learning algorithms. In the DT model, we found that IL10 expression, the patient’s age, the number of serum eosinophils, and human neutrophil elastase (HNE) count in the subepithelial area were the most important factors (Figure 2). Additionally, higher IL-10 expression, serum eosinophil count, subepithelial HNE count, and lower patient age were mainly related to a higher recurrence tendency. Specifically, on leaf no. 9, we found that patients with >41.2 serum eosinophil and >1.866 levels of IL10 showed poor surgical outcomes (approximately 70%). Moreover, leaf no. 8 revealed that patients with serum eosinophils less than 41.2 who showed >44 HNE-positive cells in the subepithelial area, and >1.866 levels of IL10 would have poor surgical outcomes (100%).

**Figure 3:**
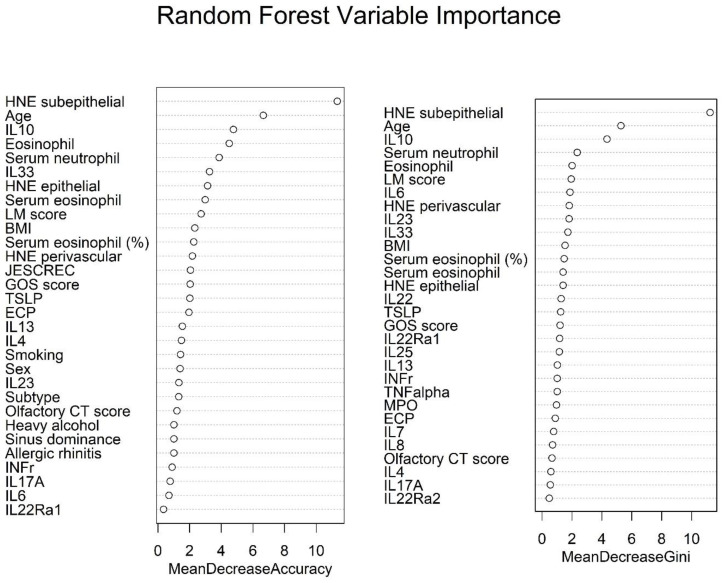
Result of the random forest model for the evaluation of surgical outcomes in patients with chronic rhinosinusitis with nasal polyps. Variable importance in terms of mean decrease in accuracy (L) and mean decrease in Gini (R) in the random forest classification. BMI—body mass index; CT—computed tomography; ECP—eosinophil cationic protein; GOS—global osteitis score; HNE—human neutrophil elastase; IL—interleukin; INF—interferon; JESCREC—Japanese Epidemiological Survey of Refractory Eosinophilic Chronic Rhinosinusitis; LM—Lund-Mackay; MPO—myeloperoxidase; TNFɑ—tumor necrosis factor; TSLP—thymic stromal lymphopoietin.

The random forest algorithm was employed as an ensemble to enhance the predictive value of the DT (Figure 3). The random forest algorithm has two methods for determining the important variables. First, the important variable was determined as the extent of the decrease in accuracy, followed by the removal of variables from the model. Second, when a variable was added to the model, the important variables were determined as the extent of the decrease in the Gini coefficient. First, we found that the top five variables in the random forest model according to the mean decrease in accuracy were (1) the HNE subepithelial number, (2) age, (3) IL10, (4) tissue eosinophil number, and (5) serum neutrophil count. Next, we investigated the Gini index, which is a measurement of this model error. This model showed the top five variables in the following order: (1) HNE subepithelial number, (2) age, (3) IL10, (4) serum neutrophil count, and (5) tissue eosinophil number. Collectively, the random forest algorithm revealed the importance of neutrophil numbers in both the tissue and serum in predicting surgical outcomes in patients with CRSwNP.

**Figure 4:**
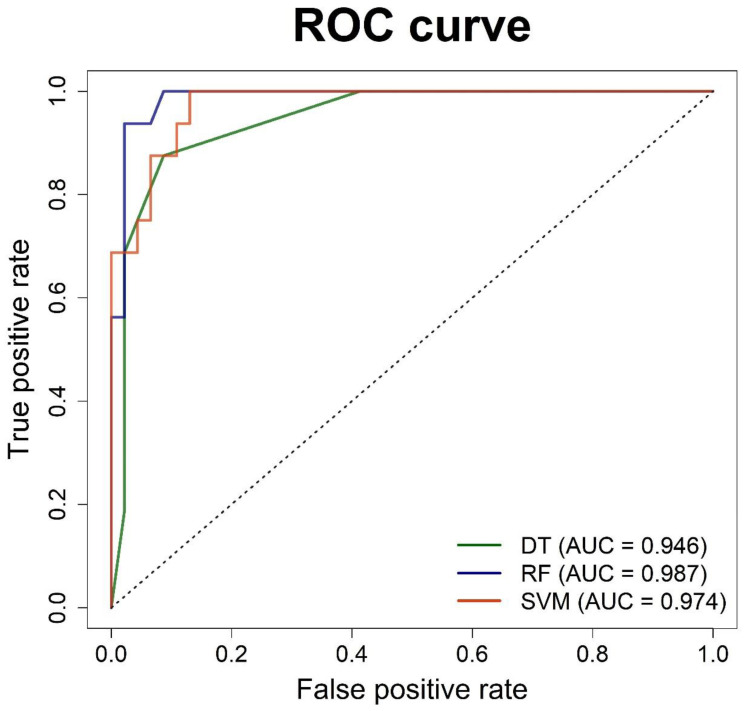
Result of the receiver operating characteristic curve of the decision tree, random forest, and support vector machine models. AUC—area under the ROC curve; DT—decision tree; RF—random forest; ROC—receiver operating characteristics.

The performance metrics of the testing data evaluated for all the classifiers are presented in Table 4. In this testing dataset, we compared several metrics and selected the best-performing classifier based on the F1 score and AUC value. Thus, our analysis revealed that the random forest algorithm had the highest F1 score and AUC. The performance of the three machine-learning algorithms is displayed in the ROC curve in Figure 4.

## 4. Discussion

Although CRSwNP inflammation is highly heterogeneous, the type 2 immune response combined with increased eosinophilic infiltration is clearly associated with refractoriness and comorbidities. Thus, in real-world clinics, physicians often encounter patients with CRSwNP who experience recurrence following sinus surgery or need repeated oral corticosteroid therapy for disease management. Recently, one study suggested that neutrophils were not only typically predominant in patients with CRSsNP but also played a major pathologic role in refractoriness in patients with CRSwNP [9]. Similarly, we found that neutrophilic inflammation may play an important role in the refractoriness of surgical patients with CRSwNP using machine learning. Although the exact mechanism of neutrophils in refractoriness could not be determined, our study revealed that age, IL10, tissue eosinophils, and neutrophils were major factors affecting surgical outcomes in patients with CRSwNP. Interestingly and notably, our machine-learning model concluded that the number of subepithelial HNE-positive cells was the most important factor in the prediction of surgical outcomes in patients with CRSwNP. Consistent with our findings, a previous study showed that the number of subepithelial HNE-positive cells was associated with increased Ki-67 expression and poor surgical outcomes in patients with CRSwNP [12]. Moreover, another study showed that HNE^+^ neutrophils were a risk factor for refractory CRSwNP in an Asian population [11].

Activated neutrophils are reportedly related to the production of major biological mediators in both innate and adaptive immune responses [16]. The increased infiltration of neutrophils in patients with CRS has been linked to a poor corticosteroid response and disease prognosis [17]. Previously, a cluster analysis study revealed that one CRS group showed only increased neutrophil markers without other elevated immune responses [18]. Additionally, several prior studies have reported that neutrophilic inflammation was elevated in patients with difficult-to-treat CRS and that the increased presence of neutrophils in the subepithelial regions of NP was associated with the severe refractoriness of CRS [9,10,11,12]. Interestingly, recent studies have suggested that CLCs can modulate neutrophilic inflammation and increase neutrophil infiltration correlates significantly with severe eosinophilia markers in patients with severe type 2 CRSwNP. This indicates that in NP tissues, eosinophil extracellular cells trap cell death-induced CLC deposition, and this deposit could initiate and maintain neutrophilic inflammation in patients with CRSwNP [19,20,21]. Moreover, multiple studies have demonstrated that elevated matrix metalloproteinase 9 expression, which produces neutrophils in the NP tissue, is strongly associated with poor wound healing and tissue regeneration following endoscopic sinus surgery in patients with CRSwNP [22,23]. Collectively, all prior reports support our main findings obtained from the machine-learning algorithm. However, contrary to previous research results, we demonstrated the hierarchy of risk factors for the recurrence of CRSwNP in patients following sinus surgery based on machine-learning modeling.

To date, studies on machine learning in CRS have been conducted; however, studies remain limited. Some studies have investigated the predictive factors for the recurrence of CRSwNP [12,24], while others have tested machine learning for the discrimination between eosinophilic and non-eosinophilic CRS [25,26]. Unlike these previous studies, our study showed that factors such as demographics, clinical variables, and inflammatory profiles effectively predicted the recurrence of CRSwNP in patients following sinus surgery. However, our study has certain limitations. First, we did not investigate the underlying pathophysiological mechanism. Thus, the role of tissue neutrophilia on the refractoriness of CRSwNP remains unclear. Second, these data were obtained from only one center; thus, a selection bias may exist in this study. To overcome this issue, multicenter studies should be conducted. Finally, certain important variables that might have affected our findings were not included.

## 5. Conclusions

The present study investigated the factors predicting the recurrence of surgical CRSwNP using a machine-learning algorithm. These machine-learning models demonstrate that neutrophilic inflammation may play an important role in the refractoriness of CRSwNP. Specifically, the random forest model suggested that subepithelial neutrophil infiltration plays a very important pathological role in the surgical outcomes of patients with CRSwNP. Therefore, clinicians should consider the possibility of poorer treatment outcomes when CRSwNP displays higher subepithelial neutrophil counts in the NP tissues.

## Figures and Tables

**Table 1:** Predictor variables and outcomes for all enrolled patients.

Chronic Rhinosinusitis with Nasal Polyp	Non-Recurrence (*n* = 156)	Recurrence (*n* = 54)	*p* Value
Endotype			0.036
Non-eosinophil	125 (80.1%)	35 (64.8%)	
Eosinophil	31 (19.9%)	19 (35.2%)	
Sex			0.033
Male	130 (83.3%)	37 (68.5%)	
Female	26 (16.7%)	17 (31.5%)	
Age	52.8 ± 13.7	41.1 ± 13.7	<0.001
Smoking			0.834
Non-smoker	99 (63.5%)	34 (63.0%)	
Ex-smoker	1 (0.6%)	0 (0.0%)	
Current	56 (35.9%)	20 (37.0%)	
Heavy alcohol			0.636
0	139 (89.1%)	50 (92.6%)	
1	17 (10.9%)	4 (7.4%)	
BMI	24.5 ± 3.4	23.9 ± 2.8	0.255
Asthma			0.029
0	147 (94.2%)	45 (83.3%)	
1	9 (5.8%)	9 (16.7%)	
Allergic rhinitis			0.140
0	95 (60.9%)	26 (48.1%)	
1	61 (39.1%)	28 (51.9%)	
LM score	14.1 ± 4.4	17.7 ± 5.1	<0.001
GOS score	11.3 ± 8.4	16.8 ± 9.2	<0.001
Sinus dominance			0.028
Ethmoid	81 (51.9%)	38 (70.4%)	
Maxillary	75 (48.1%)	16 (29.6%)	
Olfactory CT score	4.5 ± 2.5	5.5 ± 2.4	0.010
Eosinophil (tissue)	27.4 ± 47.1	49.7 ± 51.3	0.004
HNE epithelial	12.0 ± 17.5	15.2 ± 18.0	0.247
HNE subepithelial	19.9 ± 20.5	49.5 ± 31.9	<0.001
HNE perivascular	3.3 ± 5.4	6.4 ± 6.6	0.001
Serum eosinophil (%)	3.6 ± 2.5	4.6 ± 3.5	0.051
Serum eosinophil	232.4 ± 173.0	360.4 ± 265.3	0.001
Serum neutrophil	3908.9 ± 1400.8	3738.3 ± 1553.0	0.454
ECP	47.2 ± 124.5	4.9 ± 9.2	<0.001
MPO	6.4 ± 22.0	8.5 ± 15.4	0.446
IL4	22.0 ± 116.1	7.5 ± 13.7	0.130
IL5	69.1 ± 274.4	8.6 ± 15.5	0.007
IL6	97.4 ± 304.7	4.9 ± 8.9	<0.001
IL7	12.1 ± 74.2	6.0 ± 12.6	0.331
IL8	22.7 ± 105.6	14.6 ± 40.7	0.422
IL18A	29.1 ± 119.3	13.3 ± 46.7	0.170
INFγ	50.4 ± 222.7	5.0 ± 16.5	0.013
TNFɑ	64.5 ± 264.5	2.5 ± 4.6	0.004
IL10	4.8 ± 8.6	63.2 ± 167.2	0.013

Values are either expressed as *n* or mean ± standard deviation. BMI—body mass index; LM—Lund-Mackay; GOS—global osteitis score; CT—computed tomography, HNE—human neutrophil elastase; ECP—eosinophil cationic protein; MPO—myeloperoxidase; IL—interleukin; INFγ—interferon gamma; TNFɑ—tumor necrosis factor alpha.

**Table 2:** Training dataset for machine learning to predict the surgical outcome.

Chronic Rhinosinusitis with Nasal Polyp	Non-Recurrence (*n* = 110)	Recurrence (*n* = 38)	*p*
Endotype			0.171
Non-eosinophil	89 (80.9%)	26 (68.4%)	
Eosinophil	21 (19.1%)	12 (31.6%)	
Sex			0.017
Male	94 (85.5%)	25 (65.8%)	
Female	16 (14.5%)	13 (34.2%)	
Age	53.0 ± 14.3	40.3 ± 12.6	<0.001
Smoking			0.696
Non-smoker	72 (65.5%)	23 (60.5%)	
ex-smoker	1 (0.9%)	0 (0.0%)	
Current	37 (33.6%)	15 (39.5%)	
Heavy alcohol			0.827
0	98 (89.1%)	35 (92.1%)	
1	12 (10.9%)	3 (7.9%)	
BMI	24.7 ± 3.3	24.1 ± 2.9	0.301
Asthma			0.018
0	105 (95.5%)	31 (81.6%)	
1	5 (4.5%)	7 (18.4%)	
Allergic rhinitis			0.244
0	66 (60.0%)	18 (47.4%)	
1	44 (40.0%)	20 (52.6%)	
LM score	13.7 ± 4.2	17.7 ± 5.4	<0.001
GOS score	10.8 ± 7.8	16.9 ± 8.9	<0.001
Sinus dominance			0.161
Ethmoid	62 (56.4%)	27 (71.1%)	
Maxillary	48 (43.6%)	11 (28.9%)	
Olfactory CT score	4.6 ± 2.5	5.7 ± 2.3	0.018
Eosinophil (tissue)	28.7 ± 49.8	50.2 ± 55.8	0.027
HNE epithelial	10.6 ± 15.6	14.5 ± 18.2	0.202
HNE subepithelial	18.9 ± 15.4	49.8 ± 31.5	<0.001
HNE perivascular	3.4 ± 5.5	7.7 ± 7.3	0.002
Serum eosinophil (%)	3.8 ± 2.6	4.8 ± 3.8	0.151
Serum eosinophil	253.4 ± 186.7	376.4 ± 282.4	0.016
Serum neutrophil	3858.8 ± 1339.4	3814.3 ± 1714.2	0.870
ECP	56.2 ± 137.6	4.2 ± 6.3	<0.001
MPO	7.4 ± 25.6	9.2 ± 16.0	0.611
IL4	21.3 ± 110.6	8.6 ± 15.9	0.244
IL5	88.1 ± 314.5	9.3 ± 16.8	0.010
IL6	103.1 ± 303.7	5.3 ± 10.0	0.001
IL7	14.7 ± 87.5	7.6 ± 14.6	0.418
IL8	22.3 ± 94.4	8.7 ± 21.9	0.164
IL18A	26.5 ± 108.9	16.9 ± 55.3	0.489
INFγ	54.5 ± 245.7	5.8 ± 19.3	0.042
TNFɑ	69.1 ± 298.2	2.5 ± 4.2	0.021
IL10	4.6 ± 8.5	55.7 ± 145.6	0.037

Values are expressed as *n* or mean ± standard deviation. BMI—body mass index; LM—Lund-Mackay; GOS—global osteitis score; CT—computed tomography, HNE—human neutrophil elastase; ECP—eosinophil cationic protein; MPO—myeloperoxidase; IL—interleukin; INFγ—interferon gamma; TNFɑ—tumor necrosis factor alpha.

**Table 3:** Test dataset for machine learning to predict the surgical outcome.

Chronic Rhinosinusitis with Nasal Polyp	Non-Recurrence (*n* = 46)	Recurrence (*n* = 16)	*p*
Endotype			0.169
Non-eosinophil	36 (78.3%)	9 (56.2%)	
Eosinophil	10 (21.7%)	7 (43.8%)	
Sex			1.000
Male	36 (78.3%)	12 (75.0%)	
Female	10 (21.7%)	4 (25.0%)	
Age	52.4 ± 12.2	42.8 ± 16.4	0.016
Smoking			<0.001
Non-smoker	27 (58.7%)	11 (68.8%)	
ex-smoker	0 (0.0%)	0 (0.0%)	
Current	19 (41.3%)	5 (31.2%)	
Heavy alcohol			0.962
0	41 (89.1%)	15 (93.8%)	
1	5 (10.9%)	1 (6.2%)	
BMI	24.1 ± 3.6	23.6 ± 2.7	0.619
Asthma			1.000
0	42 (91.3%)	14 (87.5%)	
1	4 (8.7%)	2 (12.5%)	
Allergic rhinitis			0.535
0	29 (63.0%)	8 (50.0%)	
1	17 (37.0%)	8 (50.0%)	
LM score	15.0 ± 4.8	17.8 ± 4.5	0.041
GOS score	12.5 ± 9.6	16.6 ± 10.3	0.154
Sinus dominance			0.109
Ethmoid	19 (41.3%)	11 (68.8%)	
Maxillary	27 (58.7%)	5 (31.2%)	
Olfactory CT score	4.3 ± 2.5	5.1 ± 2.7	0.277
Eosinophil (tissue)	24.3 ± 40.3	48.4 ± 40.1	0.043
HNE epithelial	15.2 ± 21.1	16.8 ± 17.9	0.796
HNE subepithelial	22.3 ± 29.5	48.9 ± 34.0	0.004
HNE perivascular	3.1 ± 5.0	3.1 ± 2.8	0.954
Serum eosinophil (%)	3.1 ± 2.1	4.2 ± 2.7	0.086
Serum eosinophil	182.3 ± 122.7	322.2 ± 223.0	0.028
Serum neutrophil	4028.8 ± 1546.9	3557.8 ± 1106.2	0.267
ECP	25.7 ± 82.7	6.6 ± 14.0	0.139
MPO	4.0 ± 9.1	6.7 ± 14.0	0.469
IL4	23.6 ± 129.6	5.0 ± 6.0	0.338
IL5	23.6 ± 129.1	6.8 ± 12.3	0.387
IL6	83.8 ± 310.0	3.8 ± 5.3	0.087
IL7	5.9 ± 19.0	2.3 ± 3.4	0.234
IL8	23.9 ± 129.6	28.6 ± 66.1	0.853
IL18A	35.4 ± 142.1	4.7 ± 7.8	0.151
INFγ	40.7 ± 156.6	3.3 ± 5.8	0.112
TNFɑ	53.6 ± 159.5	2.7 ± 5.5	0.036
IL10	5.3 ± 9.0	81.3 ± 214.3	0.177

Values are expressed as *n* or mean ± standard deviation. BMI—body mass index; LM—Lund-Mackay; GOS—global osteitis score; CT—computed tomography, HNE—human neutrophil elastase; ECP—eosinophil cationic protein; MPO—myeloperoxidase; IL—interleukin; INFγ—interferon gamma; TNFɑ—tumor necrosis factor alpha.

**Table 4:** Summary of each machine-learning model (optimal cutoff value application).

Model	Accuracy	Sensitivity	Specificity	PPV	NPV	F1 Score	AUC	Optimal Cutoff Value
DT	0.903	0.875	0.913	0.778	0.955	0.824	0.946	0.545
RF	0.968	0.938	0.978	0.938	0.978	0.938	0.987	0.445
SVM	0.903	1.000	0.870	0.727	1.000	0.842	0.974	0.385

DT—decision tree; RF—random forest; SVM—support vector machine; PPV—positive predictive value; NPV—negative predictive value; AUC—area under the curve.

## Data Availability

The authors confirm that data supporting the findings of this study are available within the article.
